# Cause-Specific Mortality Rates Among the US Black Population

**DOI:** 10.1001/jamanetworkopen.2024.36402

**Published:** 2024-09-30

**Authors:** Adith S. Arun, César Caraballo, Mitsuaki Sawano, Yuan Lu, Rohan Khera, Clyde W. Yancy, Harlan M. Krumholz

**Affiliations:** 1 Yale School of Medicine, New Haven, Connecticut; 2 Center for Outcomes Research and Evaluation, Yale New Haven Hospital, New Haven, Connecticut; 3 Department of Internal Medicine, Yale School of Medicine, New Haven, Connecticut; 4 Section of Cardiovascular Medicine, Department of Internal Medicine, Yale School of Medicine, New Haven, Connecticut; 5 Section of Biomedical Informatics and Data Science, Yale School of Public Health, New Haven, Connecticut; 6 Department of Chronic Disease Epidemiology, Yale School of Public Health, New Haven, Connecticut; 7 Department of Health Policy and Management, Yale School of Public Health, New Haven, Connecticut; 8 Section of Health Informatics, Department of Biostatistics, Yale School of Public Health, New Haven, Connecticut; 9 Associate Editor, *JAMA*; 10 Division of Cardiology, Feinberg School of Medicine, Northwestern University, Chicago, Illinois

## Abstract

This cross-sectional study examines the specific causes of death associated with the disparities in all-cause mortality between non-Hispanic Black and non-Hispanic White populations, as well as their changes over time.

## Introduction

Understanding the disparities in all-cause mortality rates between racial and ethnic groups is crucial for addressing public health inequities in the US. Since 1999, the mortality gap between non-Hispanic Black (hereafter, *Black*) and non-Hispanic White (hereafter, *White*) populations has undergone significant fluctuations, including periods of decrease and, more recently, increase.^1^ We aim to identify the specific causes of death associated with these disparities and their change over time.

## Methods

We used the US Centers for Disease Control and Prevention (CDC) Wide-Ranging Online Data for Epidemiologic Research (WONDER) national death certificate data to collect age-adjusted mortality rates (AAMRs) per 100 000 individuals, based on the 2000 standard population, from the Black and White populations as identified by the WONDER database from 1999 through 2020, stratified by sex.^2^ We collected AAMRs for the 15 primary underlying causes of death from 1999 to 2020 among both populations separately, resulting in 18 unique causes.^2^ For 2020, we also included deaths due to COVID-19. The excess AAMR was calculated as the Black AAMR minus the White AAMR; we found that, from 1999 to 2020, these causes accounted for 83% to 88% of the overall excess AAMR. The Yale University institutional review board waived this study from review and waived participant consent because it used publicly available deidentified population-level data. Data analysis was performed with R, version 4.1.2. Data and code are publicly available.^3^ This study followed the STROBE reporting guideline.

## Results

**Figure 1.**
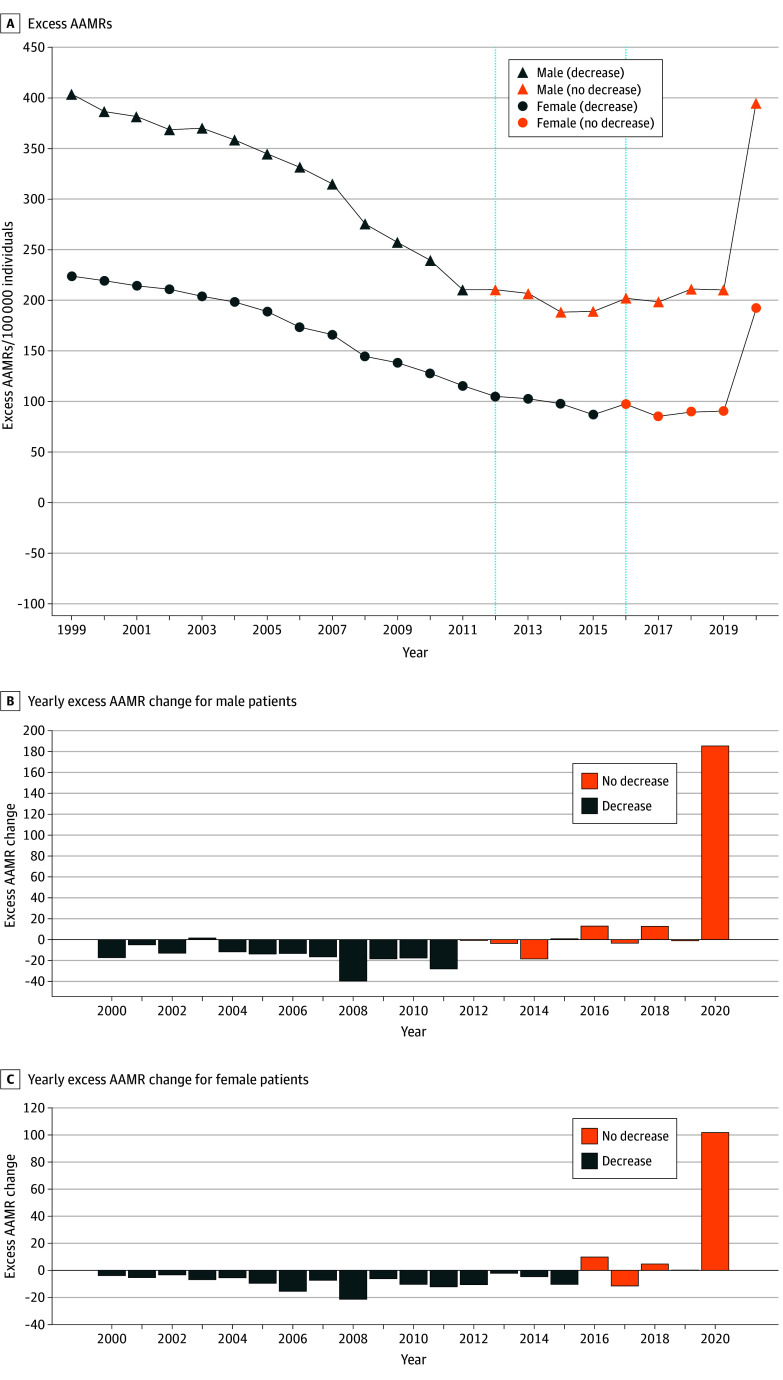
Excess Age-Adjusted Mortality Rates (AAMRs) A, Excess AAMRs per 100 000 individuals for males and females from 1999 to 2020 with years of overall excess AAMR decrease and no decrease marked. B, Yearly change in excess AAMRs for males. C, Yearly change in excess AAMRs for females. *No decrease* refers to overall periods of plateau or increase in excess AAMR.

**Figure 2.**
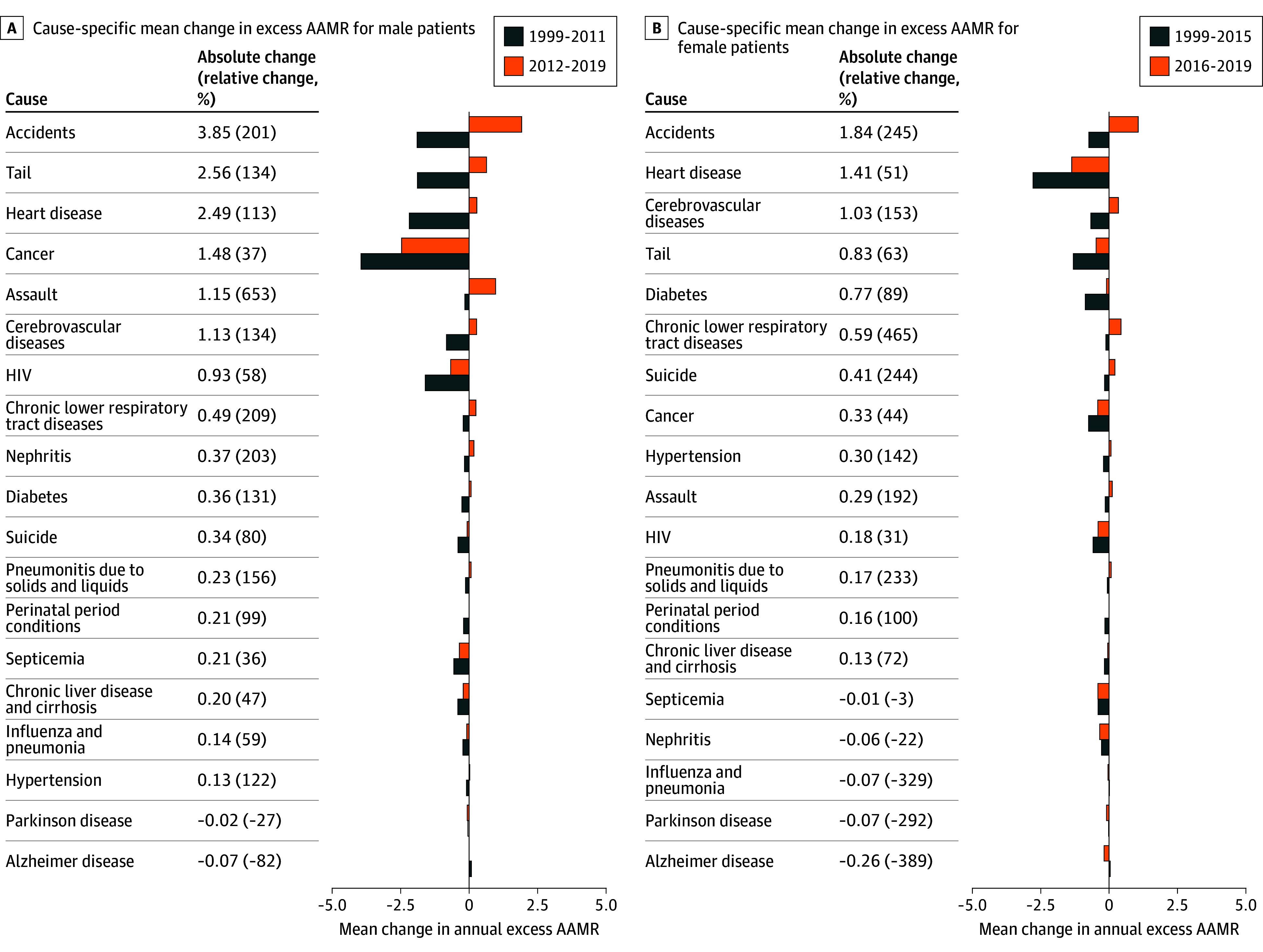
Cause-Specific Mean Change in Excess Age-Adjusted Mortality Rates (AAMRs) A, Cause-specific mean change in AAMRs for males from 1999 to 2011 and from 2012 to 2019. B, Cause-specific mean change in excess AAMRs for females from 1999 to 2015 and from 2016 to 2019. Tail indicates the approximately 15% of deaths not accounted for by the leading causes of death each year. Causes are displayed in descending order of absolute change.

From 1999 to 2020, the mean (SD) excess AAMR per 100 000 was 149 (51) and 284 (81) for females and males, respectively (Figure 1A). The excess AAMR for Black individuals decreased steadily by an annual mean of 5.7% (1999-2015) for females and 5.2% (1999-2011) for males (Figure 1B and C). Among males, absolute mean annual changes in excess AAMRs revealed that cancer (5.3% mean annual excess AAMR change), heart disease (3.5%), accidents (*ICD-10* codes V01-X59 and Y85-Y86) (60.5%), and HIV (7.0%) were primary factors associated with this decrease (Figure 2A). For females, significant absolute mean annual reductions were seen in heart disease (4.5%), diabetes (3.3%), cancer (3.7%), accidents (38.1%), and cerebrovascular diseases (2.7%) (Figure 2B).

However, there were large increases in the mean annual excess AAMR from the period of decrease to no decrease (2012-2019 for males and 2016-2019 for females) from accidents, heart disease, cerebrovascular disease, and assault (Figure 2). In 2020, the excess AAMR for males and females increased to levels last observed in 1999 and 2004, respectively (Figure 1). COVID-19 accounted for 46.1% (excess AAMR, 47 of overall 102) and 43.2% (80 of 185) of this increase in all-cause excess AAMR from 2019 to 2020 among females and males, respectively.

## Discussion

This study highlights that progress in reducing excess mortality rates among Black individuals was made primarily in reducing deaths from cancer and cardiovascular diseases among males and from cardiovascular diseases and diabetes among females. However, this progress was stalled or reversed by an increase in mortality from external causes, such as assaults and accidents, as well as a stagnation in advancements against cardiovascular diseases during periods without decrease. The COVID-19 pandemic adversely affected health outcomes among Black individuals. Marked increases in excess mortality rates in 2020 highlight the pandemic-related widening of disparities among more vulnerable subgroups, which may have long-term consequences.^4,5^

A limitation of this study is that the use of death certificate data may introduce misclassification biases, as there are inaccuracies in the reporting of cause of death and demographic characteristics. In addition, there exist differences in outcomes within CDC-categorized racial and ethnic groups (eg, US-born compared with non–US-born Black individuals) and variability in outcome trends by geography.^6^

Because race is a social construct, structural social factors contribute to progress, stagnation, and regression. There is an urgent need to determine how best to regain the progress and implement evidence-based interventions to promote health equity.

## Notes

Supplement.
Data Sharing Statement
