# Error in Figure 2

**DOI:** 10.1001/jamanetworkopen.2024.46558

**Published:** 2024-10-30

In the Research Letter titled “Cause-Specific Mortality Rates Among the US Black Population,”^1^ published September 30, 2024, the key was missing from panel A of Figure 2. This article has been corrected online.^1^
