# Supplementary material for: Cause-Specific Mortality Rates Among the US Black Population
Source: JAMA Netw Open. 2024 Sep 30;7(9):e2436402. doi: 10.1001/jamanetworkopen.2024.36402 (PMC11443349; doi:10.1001/jamanetworkopen.2024.36402)
Supplement: Supplement. — Data Sharing Statement [file jamanetwopen-e2436402-s001.pdf]

Data Sharing Statement

Arun. Cause-Specific Mortality Rates Among the US Black Population. *JAMA Netw Open*. Published September 30, 2024. doi:10.1001/jamanetworkopen.2024.36402

Data

Data available: Yes
Data types: Deidentified participant data
How to access data: All data used in this study are publicly available from https://wonder.cdc.gov. Raw data used for analysis can be found at https://github.com/aditharun/us-mort-disp-analysis/tree/main/data. All code used to analyze data and create figures are available at https://github.com/aditharun/us-mort-disp-analysis.
When available: With publication

Supporting Documents

Document types: Statistical/analytic code
How to access documents: All code used to analyze data and create figures are available at https://github.com/aditharun/us-mort-disp-analysis.
When available: With publication

Additional Information

Who can access the data: Anyone requesting the data.
Types of analyses: Per the guidelines of the CDC at https://wonder.cdc.gov.
Mechanisms of data availability: All data used in this study are publicly available from https://wonder.cdc.gov. Raw data used for analysis can be found at https://github.com/aditharun/us-mort-disp-analysis/tree/main/data. All code used to analyze data and create figures are available at https://github.com/aditharun/us-mort-disp-analysis.
